# Sargahydroquinoic Acid Suppresses Hyperpigmentation by cAMP and ERK1/2-Mediated Downregulation of MITF in α-MSH-Stimulated B16F10 Cells

**DOI:** 10.3390/foods10102254

**Published:** 2021-09-23

**Authors:** Mohammed Shariful Azam, Jae-Il Kim, Chang Geun Choi, Jinkyung Choi, Bonggi Lee, Hyeung-Rak Kim

**Affiliations:** 1 Department of Food Science and Nutrition, Pukyong National University, 45 Yongso-ro, Nam-gu, Busan 48513, Korea; bong3257@gmail.com (M.S.A.); jikim@pknu.ac.kr (J.-I.K.); choijk@pknu.ac.kr (J.C.); 2 Department of Ecological Engineering, Pukyong National University, 45 Yongso-ro, Nam-gu, Busan 48513, Korea; cgchoi@pknu.ac.kr

**Keywords:** cAMP, ERK, hyperpigmentation, melanin, MITF, sargahydroquinoic acid

## Abstract

Hyperpigmentation diseases of the skin require topical treatment with depigmenting agents. We investigated the hypopigmented mechanisms of sargahydroquinoic acid (SHQA) in alpha-melanocyte-stimulating hormone (α-MSH)-stimulated B16F10 cells. SHQA reduced cellular tyrosinase (TYR) activity and melanin content in a concentration-dependent manner and attenuated the expression of TYR and tyrosinase-related protein 1 (TRP1), along with their transcriptional regulator, microphthalmia-associated transcription factor (MITF). SHQA also suppressed α-MSH-induced cellular production of cyclic adenosine monophosphate (cAMP), which inhibited protein kinase A (PKA)-dependent cAMP-responsive element-binding protein (CREB) activation. Docking simulation data showed a potential binding affinity of SHQA to the regulatory subunit RIIβ of PKA, which may also adversely affect PKA and CREB activation. Moreover, SHQA activated ERK1/2 signaling in B16F10 cells, stimulating the proteasomal degradation of MITF. These data suggest that SHQA ameliorated hyperpigmentation in α-MSH-stimulated B16F10 cells by downregulating MITF via PKA inactivation and ERK1/2 phosphorylation, indicating that SHQA is a potent therapeutic agent against skin hyperpigmentation disorders.

## 1. Introduction

Skin pigmentation is a physiological process in which melanin pigments are synthesized in epidermal melanocytes, matured, and transferred to the neighboring keratinocytes. In melanocytes, melanin synthesis is catalyzed by tyrosinase (TYR), tyrosinase-related protein 1 (TRP1), and TRP2. Among these three enzymes, TYR is the key enzyme for melanin production. It catalyzes the hydroxylation of tyrosine to 3,4-dihydroxyphenylalanine (DOPA), and the oxidation of DOPA to dopaquinone [1,2]. In humans, melanin synthesis over basal levels usually occurs in response to solar ultraviolet radiation. There are several other factors including inflammation, hormonal factors, drug-induced causes, and cosmetics, which are associated with hyperpigmentation [3]. Hyperpigmentation occurs due to either increased numbers of melanocytes or melanogenic enzymes [4,5]. It is associated with several skin diseases such as melasma, freckles, moles, and lentigo [6,7]. Currently available commercial depigmenting agents such as kojic acid, hydroquinone [8], and arbutin [9] have satisfactory activity against hyperpigmentation disorders. However, prolonged application of hydroquinone causes irritative dermatitis, contact dermatitis, and post-inflammatory pigmentation [10]. The hydroquinone analog, arbutin is reported to be converted to hydroquinone in the human body [11]. Moreover, carcinogenic potential [12] and allergic contact dermatitis [13] caused by kojic acid, and hyperpigmentation by higher concentrations of arbutin [14] have also been reported in previous studies. Therefore, a wide range of studies has consistently focused on finding more effective and safer skin hypopigmented agents from natural sources.

In humans, melanogenesis is regulated by several signaling pathways including the cyclic adenosine monophosphate (cAMP) signaling pathway [15]. In response to alpha-melanocyte-stimulating hormone (α-MSH) or other cAMP stimulators, increased production of cellular cAMP leads to the activation of protein kinase A (PKA) via dissociating catalytic subunits from a regulatory subunit dimer. Activated catalytic subunits of PKA translocate to the nucleus and phosphorylate cAMP-responsive element-binding protein (CREB) at Ser133, which transactivates the microphthalmia-associated transcription factor (MITF) gene. MITF is the key transcriptional regulator for enhancing the tyrosinase family in melanocytes. MITF level is regulated not only by transcriptional control of CREB, but also by proteasome-dependent post-translational degradation [1]. Melanogenesis is also regulated by Akt [16] and mitogen-activated protein kinases (MAPKs) including extracellular signal-regulated kinase (ERK) [17,18,19,20], c-Jun N-terminal kinase (JNK) [21], and p38 MAPKs [22]. These MAPKs are a highly conserved family of serine/threonine kinases with diverse cellular functions [23] including the regulation of melanogenesis.

Sargahydroquinoic acid (SHQA) is a compound of the plastoquinone structural class, which was first reported from the brown alga *Sargassum serratifolium* [24]. The plastoquinone compound was previously isolated from several other Sargassum species such as *S. thunbergii* [25], *S. micracanthum* [26], *S. yezoense* [27], and *S. fallax* [28]. Previous studies have reported its anti-oxidant [25], selective vasodilatation [26], anti-diabetic, hypolipidemic [27,29,30], anti-inflammatory [31,32], and anti-skin-aging [33] activities. In our previous study, we demonstrated the hypopigmenting properties of ethanolic extract from *S. serratifolium*, and SHQA was identified as a major active component in the extract [17]. In the present study, we investigated the hypopigmented mechanisms of SHQA in α-MSH-stimulated B16F10 mouse melanoma cells.

## 2. Materials and Methods

### 2.1. Materials

The B16F10 mouse melanoma cells (ATCC CRL-6475) were collected from the American Type Culture Collection (Manassas, VA, USA). Dulbecco’s modified Eagle’s medium (DMEM) was bought from WELGENE Inc. (Gyeongsan-si, South Korea). Arbutin, α-MSH, and dimethyl sulfoxide (DMSO) were from Sigma-Aldrich Co. (St. Louis, MO, USA). Fetal bovine serum (FBS), penicillin-streptomycin, and trypsin-EDTA were purchased from GenDEPOT Inc. (Barker, TX, USA). The CellTiter 96^®^ AQueous One Solution Cell Proliferation Assay Kit was collected from Promega (Madison, WI, USA). The BCA Protein Assay Kit, Enhanced Chemiluminescence (ECL) Detection Kit, and NE-PER nuclear and cytoplasmic extraction reagents were from Thermo Scientific (Rockford, IL, USA). Thee cAMP Assay Kit was purchased from R&D Systems, Inc. (Minneapolis, MN, USA). 

### 2.2. Plant Material, Extraction, and Isolation of SHQA

**Figure 1:**
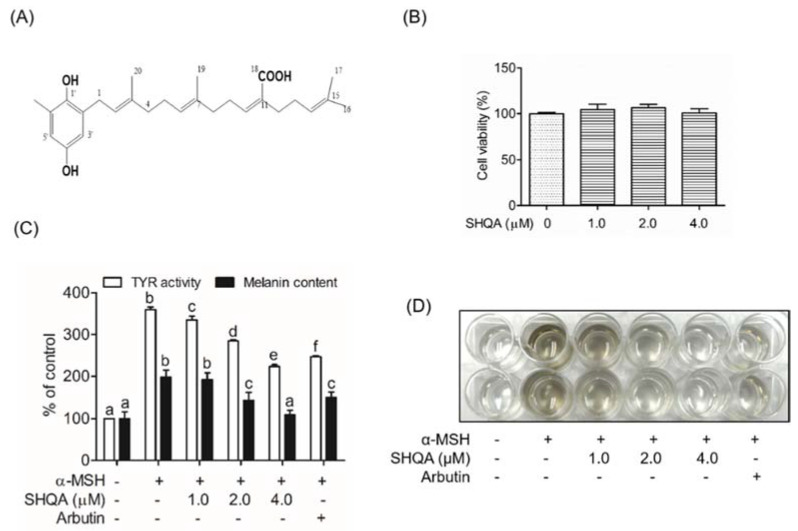
SHQA suppresses TYR activity and melanin production in B16F10 cells. (**A**) SHQA structure is isolated from *S. serratifolium*. (**B**) Cytotoxicity was measured by an MTS assay after SHQA treatment for 24 h. (**C**) After B16F10 cells were pre-treated with SHQA for 1 h, the cells were exposed to α-MSH (1.0 μM) for 72 h with and without SHQA. The cellular tyrosinase activity and melanin content was tested by measuring absorbance. (**D**) Measurement of melanin release in the culture medium after 72 h of SHQA treatment. Data are expressed as the mean ± standard deviation (SD) (*n* = 3). Different superscripts represent statistical significance at *p* < 0.05.

The brown alga *S. serratifolium* was collected from the coast of Busan, the Republic of Korea, identified and extracted with 70% ethanol, as described previously [17]. Ethanolic extract was suspended in water:ethanol (9:1, *v*/*v*) and partitioned consecutively with n-hexane, ethyl acetate, n-butanol, and water. The n-hexane fraction, which showed potent anti-melanogenic activity, was further analyzed to isolate active compounds. The n-hexane fraction (5 g/L) was separated into three fractions by a recycled HPLC system with a Luna RP-18(2) column (250 mm × 21.2 mm, 15 μm, Phenomenex, CA, USA) as described in our previous paper [31]. The chromatographic separation was repeated with an autosampler (SIL-20A) and a fraction collector (CBM-20A) and further purified by the same HPLC system with Luna RP-18 column (Luna C18(2), 250 mm × 10 mm, 5 μm). Fraction 1 was identified as SHQA (Figure 1A) using NMR spectroscopy (JNM ECP-400 spectrometer, JEOL, Japan) [31]. The elution condition of this fraction was described previously [31]. The purity of separated SHQA (>98%) was analyzed using the same HPLC system with the Luna RP-18 column (150 mm × 3 mm, 3 μm). SHQA was dissolved in the indicated concentrations in DMSO.

### 2.3. Cell Culture, Treatment, and Viability Assay

The B16F10 cells were pre-treated with SHQA for 1 h and stimulated with α-MSH for indicated periods in the presence or absence of SHQA. Cytotoxicity of SHQA was determined by the MTS assay. Briefly, 104 cells/well were seeded in a 96-well plate. After 24 h of incubation, DMEM containing 10% FBS was replaced with FBS-free DMEM and incubated for 4 h. Cells were treated with SHQA for 24 h and the culture medium was replaced with 95 µL of serum-free media and 5 µL of MTS solution. The absorbance (490 nm) was measured after 1 h of incubation.

### 2.4. Melanin Content Assay

Cellular melanin content was measured according to the previously described procedure with some modifications [34]. Briefly, B16F10 cells were incubated in a 24-well culture plate for 24 h in DMEM supplemented with 10% FBS. Cells were pre-treated with SHQA for 1 h and treated with α-MSH for 72 h in the presence or absence of SHQA. The cells were dissolved in 1 N NaOH containing 10% DMSO by boiling at 80 °C for 30 min and centrifuged at 14,000 rpm for 10 min. The supernatant absorbance was measured at 405 nm. The melanin content was measured by normalizing the absorbance with total protein levels. 

### 2.5. Intracellular cAMP Assay

The levels of cellular cAMP were measured using a commercially available cAMP ELISA Kit according to the instructions provided by the manufacturer. After the cells were pre-exposed with SHQA for 1 h, α-MSH was treated for 30 min. A cell lysis buffer in the kit was applied to the cells and supernatants were obtained to measure cAMP levels. A 96-well plate coated by streptavidin was incubated for 1 h with biotinylated cAMP-specific primary antibody. Unbound antibodies were washed three times with a wash buffer. cAMP standards, HRP-labeled cAMP, and cell lysates were added and incubated for 2 h on a shaker at room temperature. After washing, a substrate solution was added and incubated for 30 min. The reaction was stopped by adding a stop solution. The concentrations of cAMP were quantified by measuring the absorbance at 450 nm. 

### 2.6. Immunoprecipitation

B16F10 cells were treated with SHQA and lysed with a non-denaturing lysis buffer. Cell lysates were centrifuged at 14,000 rpm for 20 min at 4 °C. After washing with lysis buffer, protein G Sepharose beads were incubated with the anti-PKA (RIIβ) antibody for 3 h. The beads-anti-PKA (RIIβ) antibody complex was washed and incubated overnight with cell lysate at 4 °C with rotary agitation. The immunoprecipitated protein was eluted from the beads-anti-PKA (RIIβ) antibody complex by heating with 2× SDS-loading buffer at 95 °C for 5 min and used for western blotting.

### 2.7. Protein-Ligand Docking Simulation

The crystallographic structure of regulatory (R) subunit IIβ of human PKA (PKA-RIIβ, PDB ID: 1Cx4) was downloaded from the Research Collaboratory for Structural Bioinformatics (RCSB) Protein Data Bank (PDB) website at a resolution of 2.40 Å. The 3D structure-data file (sdf) of SHQA (CID 10202734) and bisabolangelone (BISA, CID 12300142) were downloaded from the PubChem Compound (NCBI) and converted into a protein data bank (pdb) format using Discovery Studio 4.5 (DS 4.5, http://www.accelrys.com (accessed on 3 November 2020); Accelrys, Inc., San Diego, CA, USA). Docking simulation of SHQA to the PKA-RIIβ was conducted using Auto-dock tools (ADT) 1.5.6 and Autodock vina 1.1.2. Water molecules were removed from the protein using DS 4.5 and all hydrogen atoms were added using the ADT. A Lamarkian genetic algorithm method implemented in Autodock was employed. For docking calculations, Gasteiger charges were added by default, the 66 rotatable bonds were set by the ADT, and all torsions were allowed to rotate. The cAMP binding domains of PKA-RIIβ were considered as the most suitable region for SHQA binding. The grid map was generated by the Autogrid program where the grid box size of 30 × 30 × 30 had a default spacing of 1.00 Å. The x-, y-, and z-centers were 100.516, 60.752, and 4.617, respectively. Ten binding simulation runs were performed for the SHQA-PKA binding complex. The best binding orientation was selected based on the least binding energy, which was expressed as the best binding affinity. The results were analyzed using EduPyMOL 1.7.4.5 and UCSF Chimera 1.11 (http://www.cgl.ucsf.edu/chimera/ (accessed on 3 November 2020, Pettersen et al., 2004). The hydrogen bonds and hydrophobic interacting residues were visualized by Ligplot + v.1.4.5 program.

### 2.8. Statistical Analysis

The results are shown as mean ± standard deviation (SD) of at least three independent experiments. One-way analysis of variance (ANOVA) followed by Duncan’s multiple range tests was performed using SPSS version 23 (Chicago, IL, USA). Different superscripts represent statistical significance at *p* < 0.05.

## 3. Results

### 3.1. SHQA Inhibits Cellular TYR Activity and Melanin Production in B16F10 Cells

For SHQA isolation, dried seaweed was extracted twice with 70% (*v*/*v*) ethanol. The ethanolic extract was obtained by concentration under reduced pressure. The separation and elution conditions can be referred to in [17]. The structure of SHQA is shown in Figure 1A. SHQA did not influence cell viability at concentrations up to 4.0 μM (Figure 1B). After SHQA treatment for 72 h in the presence of α-MSH, we tested cellular TYR activity and melanin content in B16F10 cells. As indicated in Figure 1C, treatment with α-MSH alone significantly increased both cellular TYR activity and melanin production (*p* < 0.05) while SHQA pre-treatment markedly reduced α-MSH-stimulated TYR activity (EC50 = 3.79 ± 0.11 μM) and melanin production (EC50 = 2.21 ± 0.47 μM) in a concentration-dependent manner. Figure 1D reproducibly demonstrates reduced levels of extracellular melanin released in the culture medium after 72 h of SHQA treatment.

### 3.2. SHQA Suppresses the Production of Melanogenic Enzymes TYR and TRP1, and Their Transcription Factor MITF

**Figure 2:**
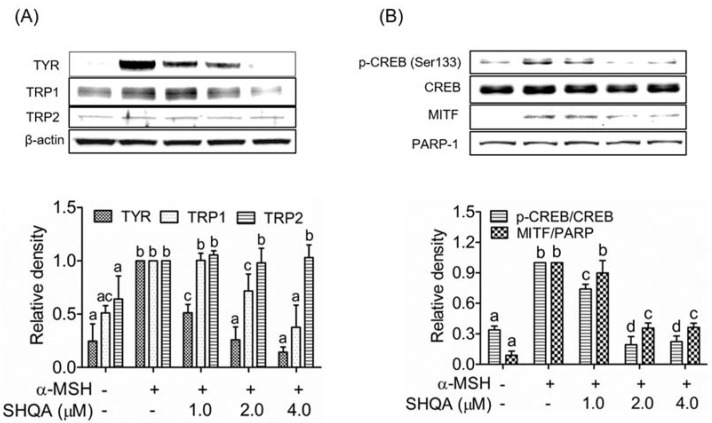
SHQA suppresses the protein levels of enzymes and transcription factors related to melanogenesis in B16F10 cells. (**A**) After pre-treatment of SHQA for 1 h, cells were treated with α-MSH (1.0 μM) for 48 h with or without SHQA. Western blotting was performed for TYR, TRP1, and TRP2. After pre-treatment of SHQA for 1 h, cells were treated with α-MSH for 6 h with or without SHQA. (**B**) Western blotting was performed for nuclear MITF and CREB. Data represent the mean ± standard deviation SD (*n* = 3). Data are expressed as the mean ± standard deviation (SD) (*n* = 3). Different superscripts represent statistical significance at *p* < 0.05.

We examined the effect of SHQA on the production of three melanogenic enzymes, TYR, TRP1, and TRP2. SHQA inhibited the production of TYR and TRP1 in a dose-dependent manner, whereas it did not affect TRP2 production (Figure 2A). We further measured the expression of their transcription factor, MITF, in the nuclear fraction of B16F10 cells. SHQA significantly downregulated MITF production in a dose-dependent manner (Figure 2B, *p* < 0.05). These results suggest that SHQA inhibited the production of TYR and TRP1 through the downregulation of MITF.

### 3.3. SHQA Inhibits CREB Activation in B16F10 Cells

We assessed whether SHQA could inhibit the phosphorylation of CREB at serine 133 (Ser133), as CREB is a key modulator of MITF production. As shown in Figure 2B, CREB phosphorylation at Ser133 was significantly increased upon stimulation with α-MSH in B16F10 cells (*p* < 0.05), while SHQA treatment for 6 h inhibited CREB phosphorylation in a dose-dependent manner. This finding indicates that SHQA suppressed TYR and TYR-1 production in B16F10 cells by inhibiting CREB-mediated MITF production.

### 3.4. SHQA Suppresses Cellular cAMP Production and PKA Activation

**Figure 3:**
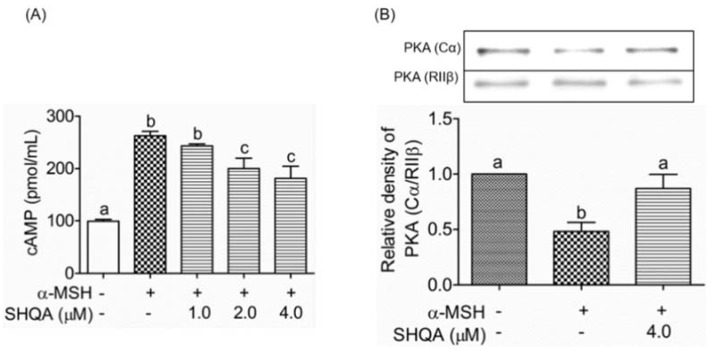
SHQA prevents cAMP production and PKA activation in B16F10 cells. (**A**) After pre-treatment of SHQA for 1 h, cells were treated with α-MSH (1.0 μM) for 30 min with or without SHQA. Intracellular cAMP concentration was measured by a cAMP ELISA kit. (**B**) Cell lysates were immunoprecipitated with anti-PKA-RIIβ antibody and PKA-Cα co-precipitation was tested by western blotting. The band images were measured by densitometry and shown as bar graphs. Data are expressed as the mean ± standard deviation (SD) (*n* = 3). Different superscripts represent statistical significance at *p* < 0.05.

Because cAMP plays a pivotal role in melanogenic signaling pathways, we examined whether SHQA influences the cellular level of cAMP. With SHQA treatment for 30 min, α-MSH-induced cAMP levels were reduced in a dose-dependent manner (Figure 3A). Next, we determined whether inhibition of cAMP by SHQA affected PKA activation. The SHQA-treated cell lysate was immunoprecipitated with an anti-PKA (RIIβ) antibody, a regulatory subunit of PKA, and the associated catalytic subunit α of PKA (PKA-Cα) coprecipitated with the RIIβ subunit was detected by western blot. As shown in Figure 3B, SHQA inhibited α-MSH-induced dissociation of the Cα subunit from the RIIβ subunit, resulting in PKA inactivation. These results suggest that SHQA suppressed intracellular cAMP production and subsequently suppressed PKA activation, leading to the CREB-mediated downregulation of MITF.

### 3.5. SHQA May Have Binding Affinity to PKA (RIIβ)

**Figure 4:**
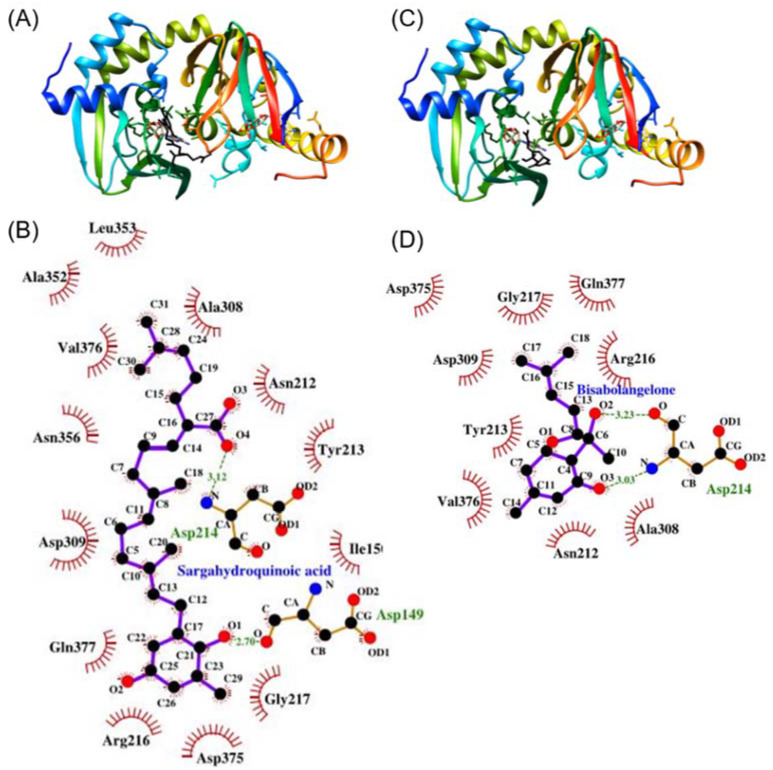
Protein-ligand docking simulation of SHQA to the cAMP binding domain of PKA. (**A**) Docking simulation of SHQA to the regulatory subunit IIβ of PKA. cAMP and SHQA are shown in the gray and black colors, respectively. (**B**) Binding mode analysis between SHQA and PKA (RIIβ). (**C**) Docking simulation of BISA and the regulatory subunit IIβ of PKA. (**D**) Binding mode analysis between BISA and PKA (RIIβ).

With the experimental finding that SHQA suppressed cAMP-induced dissociation and activation of the PKA holoenzyme, we performed protein-ligand molecular docking simulation to predict the possibility about whether SHQA may bind to the PKA-RIIβ subunit. In this simulation, BISA, a reported inhibitor of cAMP binding to the PKA-RIIβ subunit [35], was used as a standard ligand for validating docking simulation. Thus, the docking simulation between BISA and PKA was also conducted (Figure 4C). As shown in Figure 4A,B, SHQA might bind in close proximity to the amino acid residues of the cAMP binding domain of PKA-RIIβ. The predicted binding energy of SHQA with the PKA-RIIβ subunit was estimated to be −7.0 kcal/mol with two hydrogen bonds, which was lower than that of BISA with the same subunit (−6.1 kcal/mol). Both SHQA and BISA might interact with the same amino acid residues of PKA with hydrophobic interactions and SHQA might interact with an additional four amino acids (Figure 4B,D). The carboxyl group of Asp149 and the nitrogen group of Asp214 of PKA were involved in hydrogen bonding with the oxygens O1 and O4 of SHQA, respectively (Figure 4B). Although it should be proven by additional experiments including isothermal titration, our data provide the possibility that SHQA may have a higher affinity for binding to PKA than BISA. 

### 3.6. SHQA Induces Proteasome-Mediated Degradation of MITF via ERK1/2 Activation

**Figure 5:**
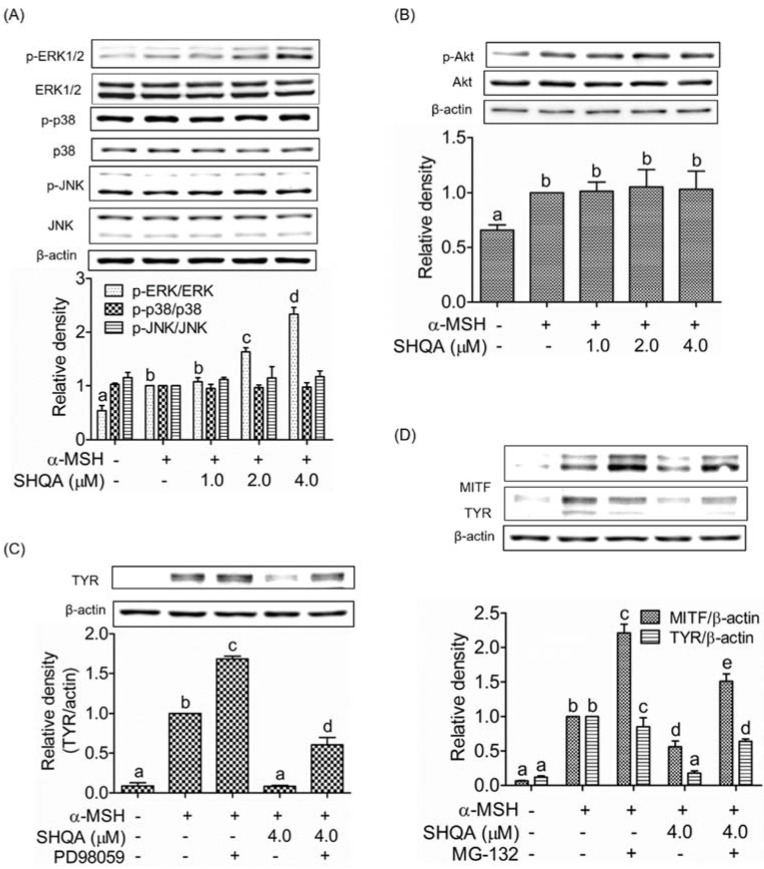
Effect of SHQA on melanogenic upstream signaling pathways in B16F10 cells. After pre-treatment of SHQA for 1 h, cells were treated with α-MSH (1.0 μM) for 1 h with or without SHQA. Western blotting was performed for (**A**) MAPKs and (**B**) AKT. (**C**) After pre-treatment of SHQA for 1 h with or without PD98059 (15.0 μM), cells were treated with α-MSH for 48 h. Western blotting was performed to detect tyrosinase. (**D**) After pre-treatment of SHQA for 1 h with or without MG-132 (70 nM), cells were treated with α-MSH for 48 h. Western blotting was performed for MITF and tyrosinase. The band images were measured by densitometry and shown as bar graphs. Data are expressed as the mean ± standard deviation (SD) (*n* = 3). Different superscripts represent statistical significance at *p* < 0.05.

We determined the effects of SHQA on the phosphorylation of MAPKs and Akt to find any additional signaling pathways involved in the hypopigmenting mechanism of SHQA in B16F10 cells. SHQA significantly phosphorylated ERK1/2 in a dose-dependent manner, which was responsible for the post-translational degradation of MITF (Figure 5A, *p* < 0.05) whereas SHQA did not change the phosphorylation of Akt, JNK, and p38 MAPK (Figure 5A,B). We further confirmed the involvement of ERK1/2 signaling in the hypopigmenting action of SHQA using the ERK inhibitor PD98059. As shown in Figure 5C, SHQA markedly suppressed α-MSH-stimulated TYR production, whereas co-treatment with the ERK inhibitor attenuated SHQA-mediated TYR inhibition, indicating that ERK phosphorylation is partially associated with TYR expression. We further investigated whether ERK1/2 phosphorylation has any effect on MITF degradation. Thus, we assessed the production of MITF and TYR in the presence of MG-132, a proteasome inhibitor. As shown in Figure 5D, SHQA significantly inhibited the α-MSH-stimulated production of both MITF and TYR (*p* < 0.05). The suppressive effect of SHQA on MITF and TYR production was abolished with the proteasome inhibitor, indicating that SHQA induces proteasome-mediated MITF degradation. These results demonstrate that ERK1/2 phosphorylation by SHQA resulted in the downregulation of TYR via proteasome-mediated degradation of MITF.

**Figure 6:**
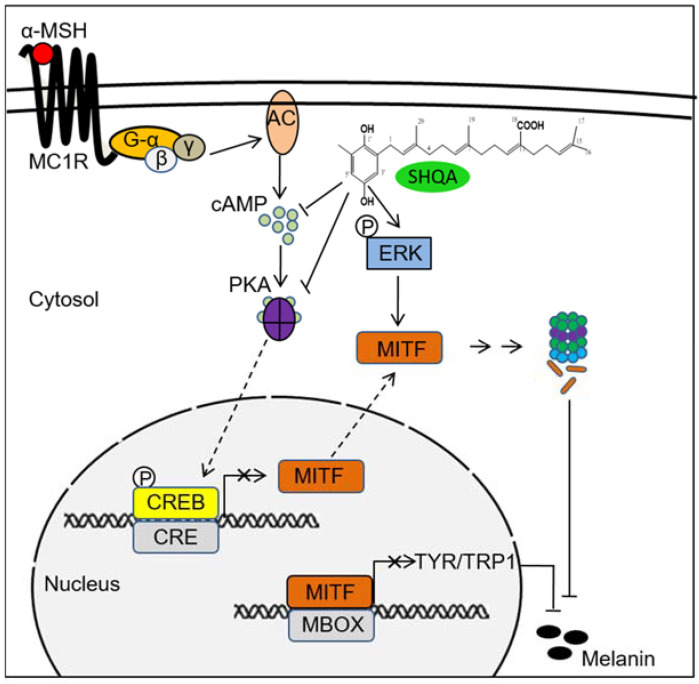
Hypothetical mechanism showing the anti-melanogenic effect of SHQA in α-MSH-stimulated B16F10 cells. The illustration represents the MITF downregulation by SHQA via the inhibition of cAMP- and PKA-dependent CREB activation and the proteasomal degradation of MITF via the activation of ERK1/2 signaling. Downregulation of MITF leads to the suppressed production of TYR and TRP1 enzymes, leading to the suppression of melanin production.

Based on our current results, we propose dual mechanisms of SHQA on the inhibition of α-MSH-induced hyperpigmentation in B16F10 melanoma cells (Figure 6). SHQA inhibited the production of MITF through cAMP- and PKA-dependent CREB activation. Additionally, SHQA induced proteasomal degradation of MITF by ERK1/2 activation. As a result, downregulated MITF resulted in the reduced production of TYR and TRP1, leading to the suppressed melanin production in α-MSH-treated B16F10 cells.

## 4. Discussion

In the previous study, we demonstrated hypopigmenting mechanisms of the ethanolic extract from *S. serratifolium* using α-MSH-stimulated melanoma cells, and we found that SHQA was the major active compound in the ethanolic extract [17]. Thus, we isolated SHQA from the ethanolic extract and its hypopigmenting mechanisms were investigated using α-MSH-stimulated melanocytes. In this study, we found that SHQA inhibited intracellular TYR activity and melanin production in a dose-dependent manner and induced downregulation of melanogenic enzymes via reduced production of MITF in B16F10 cells. 

In melanocytes, melanin synthesis is directly catalyzed by the melanogenic enzymes TYR, TRP1, and TRP2 [5]. Among these three enzymes, TYR catalyzes the first two rate-limiting steps: the hydroxylation of tyrosine to 3, 4-dihydroxyphenylalanine (DOPA), and the oxidation of DOPA to DOPAquinone [5]. The DOPAquinone is further catalyzed by TRP1 and TRP2 to produce eumelanin. DOPAquinone can also be converted to phaeomelanin by nonenzymatic polymerization. Therefore, TYR is a key enzyme for the synthesis of both eumelanin and phaeomelanin, whereas TRP1 and TRP2 are recognized as crucial factors for the synthesis of eumelanin [5,36]. Although there is sequence similarity with the tyrosinase family, the mechanism of TYP2 expression may be substantially different from that of either TRP-1 and TYR [37]. The expression of TRP2 is found in migratory melanocytes and melanocyte precursors earlier than the expression of TYR and TRP1 [37]. In this study, we found that SHQA inhibited the expression of TYR and TRP1, but not TRP2, which resulted in the suppressed production of melanin in α-MSH-stimulated B16F10 cells. Moreover, inhibition of cellular TYR activity by SHQA would be contributed to reduced melanin production (Figure 1C).

MITF plays a central role in skin pigmentation by upregulating melanogenic enzymes. It not only regulates pigmentation-related gene expression, but is also essential for proliferation, differentiation, and survival of melanocytes [1]. Overproduction of MITF in melanocytes results in hyperpigmentation with uncontrolled TYR production. The expression of MITF in melanocytes is regulated by at least four transcription factors such as CREB, lymphoid-enhancing factor 1, sex-determining region Y family member, and paired box-containing transcription factor [1]. Among them, CREB has been recognized as one of the key transcription factors for the regulation of MITF in melanocytes [1]. CREB phosphorylation at Ser133 is required for its activation [38] and DNA-binding activity [39]. Therefore, inhibition of CREB phosphorylation at Ser133 could be a good strategy to inhibit MITF expression [35,40]. In the present study, we found that SHQA inhibited CREB phosphorylation and led to the downregulation of MITF. MITF downregulation caused reduced production of TYR and TRP1 and eventually suppresses melanogenesis in B16F10 cells. 

Cellular cAMP is considered to be the second messenger to stimulate PKA-mediated CREB activation [3,5]. Upon α-MSH binding to the melanocortin-1 receptor, overproduced cAMP cooperatively binds to two binding domains on each regulatory subunit of PKA that induces an obligatory conformational change of the regulatory subunit to release the catalytic subunits [41]. The released catalytic subunits of PKA translocate to the nucleus to phosphorylate their substrate CREB, which is responsible for the transactivation of MITF [5]. Thus, inhibiting PKA activation by attenuating cAMP production could be a potential therapeutic target against hyperpigmentation disorders. We found that SHQA inhibited cAMP production, leading to the inhibition of PKA activation via dissociation of the catalytic subunit Cα from the regulatory subunit RIIβ. Inhibiting PKA activation by direct blocking of the dissociation of catalytic subunits from regulatory subunits is also a therapeutic target against hyperpigmentation disorders [35,40]. BISA and diphenylmethylene hydrazinecarbothioamide are competitive inhibitors of cAMP binding to the regulatory subunit RIIβ of PKA, thus interfering with the cAMP signaling by inhibiting the dissociation between catalytic and regulatory subunits [35,40]. In our study, SHQA showed a higher binding affinity to PKA (RIIβ) compared to that of BISA because of the involvement of more interacting residues as determined by the molecular docking simulation. These results indicate that SHQA inhibited melanin production by interfering with the cAMP signaling pathway via binding of cAMP to the cAMP binding domain of PKA.

In melanocytes, MITF level is regulated by transcriptional activation of CREB as well as by ubiquitin-dependent proteasomal degradation [1,42]. Accumulated findings suggest that MAPKs [19,21,22,43] are important players in MITF degradation. In particular, phosphorylation of ERK1/2 suppressed melanogenesis by post-translational degradation of MITF [44,45]. Phosphorylated ERK1/2 induces MITF phosphorylation, leading to proteasomal degradation of MITF [18,43,46]. In this study, the involvement of ERK signaling in the anti-melanogenic activity of SHQA was confirmed by treating cells with the ERK inhibitor PD98059 and it reversed the suppressive effect of SHQA on TYR expression. Moreover, treatment with the proteasome inhibitor MG-132 abrogated SHQA-induced suppression of MITF and TYR expression, suggesting that ERK1/2-induced proteasomal degradation of MITF largely contributes to the hypopigmenting properties of SHQA.

Although we showed that SHQA may bind to the regulatory subunit RIIβ of PKA by protein-ligand docking simulation, it should be noted that the docking simulation data in our study included limitations. The primary limitation of molecular docking is because of the shortage of confidence in the ability of scoring functions to provide precise binding energies. It results from the fact that some intermolecular interaction terms may be hardly predicted precisely such as solvation effect and entropy change [47,48]. Furthermore, some intermolecular interactions may not be considered in scoring functions, which have been proven to be of significance. Isothermal titration experiments will be necessary to measure reactions between SHQA and the regulatory subunit RIIβ of PKA including binding affinity, stoichiometry, entropy, and enthalpy of the binding reaction.

Collectively, our data suggest that SHQA is an efficient hypopigmenting compound from marine brown algae. Hypopigmenting mechanisms of SHQA are primarily mediated by suppressing the cAMP/CREB/MITF pathway and activating the ERK1/2 signaling pathway in which both pathways downregulated MITF production. Finally, this study suggests a potential application of SHQA in the treatment of skin hyperpigmentation disorders and as a cosmetic component to improve skin whitening.

## Data Availability

The datasets generated for this study are available on request to the corresponding author.
